# Detection of *Anopheles rivulorum*-like, a member of the *Anopheles funestus* group, in South Africa

**DOI:** 10.1186/s12936-018-2353-y

**Published:** 2018-05-15

**Authors:** Joel Mouatcho, Anthony J. Cornel, Yael Dahan-Moss, Lizette L. Koekemoer, Maureen Coetzee, Leo Braack

**Affiliations:** 1 UP Institute for Sustainable Malaria Control & MRC Collaborating Centre for Malaria Research, University of Pretoria, Pretoria, South Africa; 2 Department of Entomology and Nematology, University of California, Davis, CA 95616 USA; 3 Wits Research Institute for Malaria, MRC Collaborating Centre for Multi-disciplinary Research on Malaria, School of Pathology, Faculty of Health Sciences, University of the Witwatersrand, Johannesburg, South Africa; 4 Centre for Emerging Zoonotic and Parasitic Diseases, National Institute for Communicable Diseases, Johannesburg, South Africa

**Keywords:** *Anopheles funestus*, *An. rivulorum*-like, Malaria, Vector distribution, Mosquitoes, South Africa

## Abstract

**Background:**

The *Anopheles gambiae* sensu lato (s.l.) and *Anopheles funestus* s.l. species complexes contain the most important malaria vectors in Africa. Within the *An. funestus* group of at least 11 African species, the vector status of all but the nominal species *An. funestus* appears poorly investigated, although evidence exists that *Anopheles rivulorum* and *Anopheles vaneedeni* may play minor roles. A new species, *An. rivulorum*-like, was described from Burkina Faso in 2000 and subsequently also found in Cameroon and Zambia. This is the first paper reporting the presence of this species in South Africa, thereby significantly extending its known range.

**Methods:**

Mosquitoes were collected using dry-ice baited net traps and CDC light traps in the Kruger National Park, South Africa. Sixty-four *An. funestus* s.l. among an overall 844 mosquitoes were captured and identified to species level using the polymerase chain reaction assay. All samples were also analysed for the presence of *Plasmodium falciparum* circumsporozoite protein using the enzyme-linked-immunosorbent assay.

**Results:**

Four members of the *An. funestus* group were identified: *An. rivulorum*-like (n = 49), *An. rivulorum* (n = 11), *Anopheles parensis* (n = 2) and *Anopheles leesoni* (n = 1). One mosquito could not be identified. No evidence of *P. falciparum* was detected in any of the specimens.

**Conclusion:**

This is the first report of *An. rivulorum*-like south of Zambia, and essentially extends the range of this species from West Africa down to South Africa. Given the continental-scale drive towards malaria elimination and the challenges faced by countries in the elimination phase to understand and resolve residual transmission, efforts should be directed towards determining the largely unknown malaria vector potential of members of the *An. funestus* group and other potential secondary vectors.

## Background

The most important and widespread vectors of malaria in Africa are members of two species complexes: *Anopheles gambiae* sensu lato (s.l.) with at least eight sibling species [1], and *Anopheles funestus* s.l. with 11 African species [2, 3]. Adult members of these two complexes are difficult or impossible to separate morphologically [4–6], requiring molecular techniques for reliable identification [1, 7–9]. Species within both complexes vary greatly in feeding behaviour, host preferences and in particular their efficiency as vectors of malaria [4–6, 10, 11].

Within the *An. funestus* group, *An. funestus* sensu stricto (s.s.) is widely acknowledged as one of the three most efficient and important vectors of malaria in Africa [4, 5, 12]. As for other members of the *An. funestus* group, some reports indicate that *An. rivulorum* may be involved in malaria transmission in some situations [13–15], and *Plasmodium falciparum* has been reported from *Anopheles parensis* and *Anopheles leesoni* [14]. *Anopheles vaneedeni* has been experimentally infected with *P. falciparum* [16], and *P. falciparum* has recently been isolated in natural populations of this species in South Africa [11]. No reports of any involvement in malaria transmission for the remaining members of the *An. funestus* group were found. Because of the close morphological similarity but very different malaria transmission capacities of these species, it is important to know which occur in a particular area as this influences malaria vector control decisions and operations, vital commitments in all African countries where financial and related malaria control resources are limited.

The Afrotropical members of the *An. funestus* group can be categorized into three subgroups (Table 1), of which the Rivulorum Subgroup with four species is of relevance here. Aside from *Anopheles rivulorum*, *Anopheles brucei* and *Anopheles fuscivenosus*, this Subgroup also includes *An. rivulorum*-like that was first described from Burkina Faso as a cryptic but unnamed taxon [17], subsequently called “*An. rivulorum*-like” by Cohuet et al. [9] based on specimens caught in Cameroon. Later, Norris and Norris [18] reported *An. rivulorum*-like also present in Zambia. Its presence has not been recorded elsewhere.

**Table 1:** Afrotropical species members and key attributes of the *Anopheles funestus* Group

Subgroup [28]	Species	Distribution (based on [4, 5, 9, 12, 28, 29])	Malaria vector status (based on [5, 28, 29])
Funestus	*An. funestus*	Sub-Saharan Africa	Major
Funestus	*An. funestus*-like	Malawi	Unknown
Funestus	*An. aruni*	East Africa	Unknown
Funestus	*An. confusus*	East Africa	Unknown
Funestus	*An. parensis*	East and southern Africa	Possible [14]
Funestus	*An. vaneedeni*	South Africa	Confirmed [11]
Funestus	*An. longipalpis type C*	Zambia	Unknown
Minimus	*An. leesoni*	Sub-Saharan Africa	Possible [14]
Minimus	*An. longipalpis type A*	South Africa	Unknown
Rivulorum	*An. rivulorum*	Sub-Saharan Africa	Minor to potentially locally important [13–15]
Rivulorum	*An. rivulorum*-like	Burkina Faso, Cameroon, Zambia, and now also South Africa.	Unknown
Rivulorum	*An. brucei*	Nigeria	Unknown
Rivulorum	*An. fuscivenosus*	Northern parts of southern Africa	Unknown

In this paper, findings indicating populations of *An. rivulorum*-like in two localities within the Kruger National Park, north-eastern South Africa, are presented, thereby expanding the known range of this species from West Africa (Burkina Faso, Cameroon) and Zambia down to South Africa, the southern limit of malaria transmission in Africa.

## Methods

### Study area

**Fig. 1:**
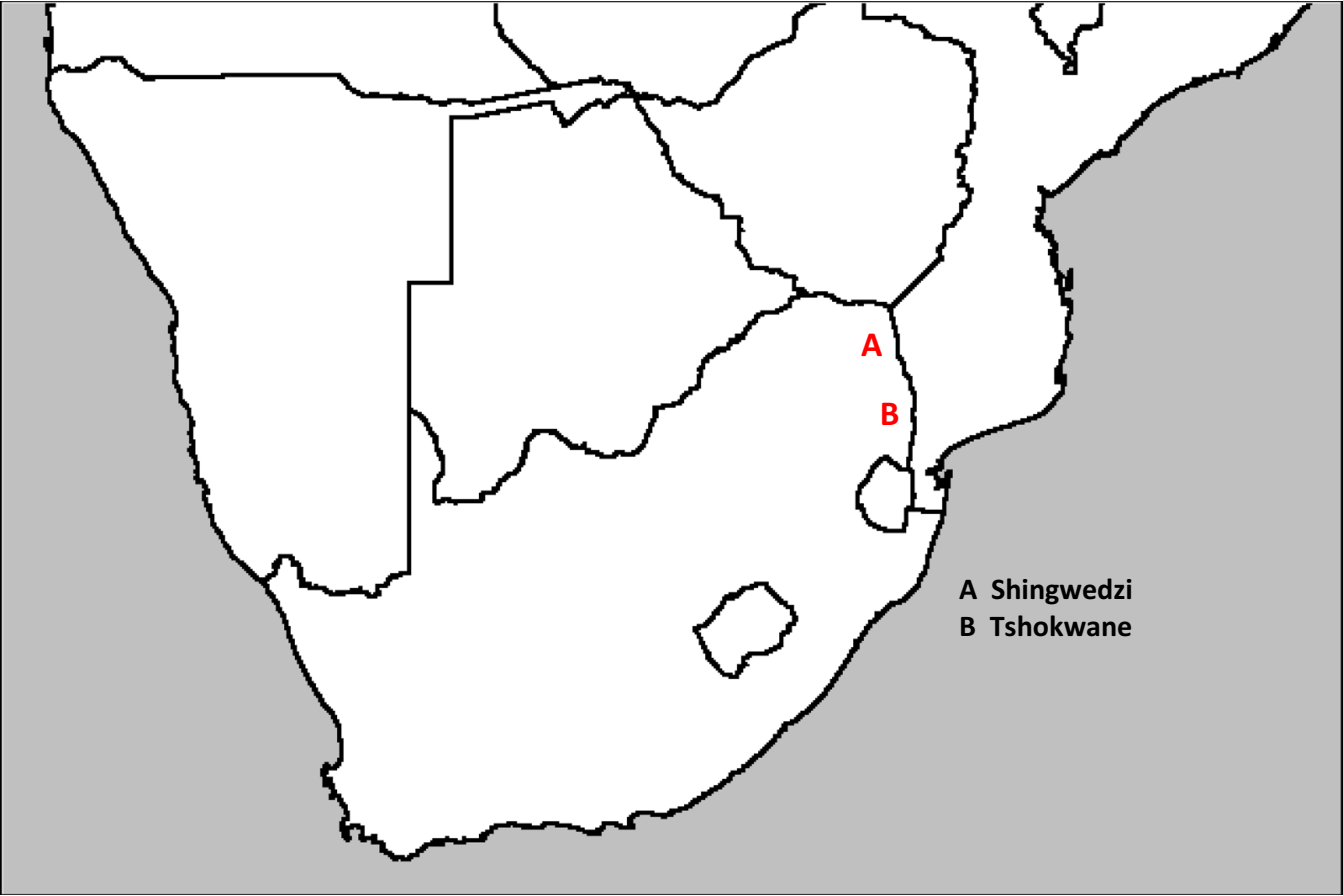
Map showing geographic spread of sampling sites

Mosquito collections were undertaken in March 2015 in the Kruger National Park as part of a broader study on mosquito distribution and abundance in South Africa. The Kruger National Park (KNP) is located in the north-eastern corner of South Africa, bordering Mozambique to the east and Zimbabwe to the north. The specific localities where traps were placed within this ca 19,000 km^2^ National Park are the Shingwedzi River within 20 km west and east of Shingwedzi Camp (S23° 06.656′, E31° 27.419′), and in the mostly dry bed of the Nwaswitsontso River within 500 metres of the Tshokwane Picnic Site (S24° 47.121′, E31° 51.283′) (Fig. 1). Shingwedzi is in the northern part of the KNP, within the Limpopo Province of South Africa, and Tshokwane in the Mpumalanga Province, both these Provinces being malaria endemic. All sites where anophelines were collected had standing pools of water in the river-bed with an abundant growth of aquatic plants floating on the surface.

### Mosquito collections

Mosquito net traps [19] and CDC light traps [20] baited with dry-ice were used to collect adult mosquitoes. All *Anopheles* mosquitoes were identified using morphological keys [5], and members of the *An. gambiae* and *An. funestus* groups were individually placed in silica gel tubes for subsequent PCR identification. Sixty-four *An. funestus* s.l. were captured, representing 7.6% of the total 844 mosquitoes collected.

### Identification of *Anopheles funestus* members

Genomic DNA from *Anopheles* mosquitoes were individually extracted from two legs/two wings in 30 µl sodium chloride-Tris–EDTA (STE) buffer (1 M NaCl, 1 M Tris–HCl and 0.5 M EDTA). Specimens were ground in STE buffer and incubated for 12 min at 94 °C. The extracted DNA was assayed by PCR using species-specific primers to separate six members of the *An. funestus* group including *An. rivulorum*-like [8, 9]. *Anopheles funestus* species-specific PCR was done according to the protocol described by Koekemoer et al. [8]. Positive and negative controls were added with the unidentified *Anopheles* specimens in each PCR reaction. Positive controls included the following species of the *An. funestus* group: *An. funestus*, *An. leesoni*, *An. parensis*, *An. rivulorum* and *An. vaneedeni*. The negative controls included a PCR mastermix without template as well as a DNA extraction negative control (DNA extraction performed without mosquito sample).

Following amplification, 10 µl of the PCR products were fragment-size separated through a 2.5% agarose gel stained with ethidium bromide and were visualized on a UV trans-illuminator. To confirm the identity of the unexpected *An. rivulorum*-like specimens, amplicons from these specimens were excised from the gel, purified using Wizard^®^SV (Promega) and sequenced using the ABI 3130 Genetic Analyser by Macrogen Inc.

### Confirmation of *An. rivulorum*-like species identification

DNA was extracted from three specimens that were suspected to be *An. rivulorum*-like, using prepGEM^®^ DNA Extraction Kits (ZyGEM™). The internal transcribed spacer region 2 (ITS2), which is the noncoding region between the 5.8S and 28S coding region of the specimens was amplified by PCR according to the protocol by Koekemoer et al. [8].

Subsequently, the PCR products of the ITS2 region from the three specimens were purified and sequenced by Macrogen Inc. The chromatograms of the sequences were analysed by using BioEdit version 7.2.5 [21]. The resulting sequences of the 3 specimens were compared to each other by using the Muscle multiple sequence alignment [22] and a consensus sequence was established. Subsequently, the Emboss Needle pairwise sequence alignment tool [23] was used to compare the consensus sequence with *An. rivulorum*-like sequences with the following GenBank accession numbers: KR014818 [24], JN994147 [18] and AF210725 [17] as well as an *An. rivulorum* sequence GenBank accession number JN994148 [18].

### *Plasmodium falciparum* detection

The head and thorax of each female mosquito was subjected to indirect enzyme-linked immunosorbent assay (ELISA) for presence of *P. falciparum* circumsporozoite protein (CSP) using monoclonal antibodies 2A10 as described by Wirtz et al. [25]. One positive control and seven negative controls were added to each microtitre plate. The positive control consisted of a synthetic peptide standardized against the human malaria parasite, *P. falciparum*. Negative controls were unfed *An. funestus* s.s. from laboratory colonies maintained by the Vector Control Reference Laboratory, National Institute for Communicable Diseases, Johannesburg. Absorbance was measured at 405 nm using a microtitre plate reader. The cut-off value for positive specimens was taken as twice the mean value of the negative controls.

## Results

**Fig. 2:**
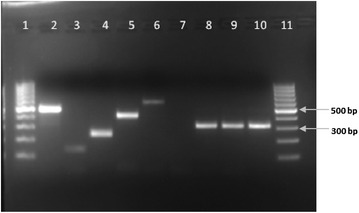
*An. funestus* group PCR confirmed that the amplified PCR fragment of the Shingwedzi specimens corresponds to the *An. rivulorum*-like size fragment of 313 bp. Lane 1 and 11: 100 bp DNA ladder; lane 2: *An. funestus* positive control; lane 3: *An. leesoni* positive control; lane 4: *An. parensis* positive control; lane 5: *An. rivulorum* positive control; lane 6: *An. vaneedeni* positive control; lane 7: negative control; lanes 8–10: three specimens from Shingwedzi

Of the 64 *Anopheles funestus* s.l. identified morphologically, 63 were identified to species level by PCR as *An. rivulorum*-like (n = 49), *An. rivulorum* (n = 11), *An. parensis* (n = 2) and *An. leesoni* (n = 1). One sample failed to amplify, which may be due to human error, DNA degradation or morphological miss-identification. The PCR of the *An. rivulorum*-like specimens produced a diagnostic 313 bp fragment [9] (Fig. 2). The species-specific PCR was repeated twice to confirm the results.

**Fig. 3:**
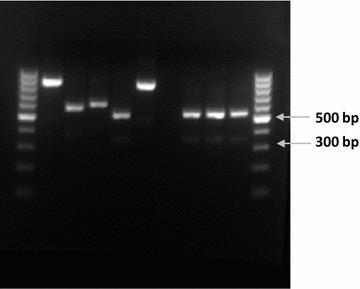
PCR of the ITS2 region of members of the *An. funestus* group and three examples of the Shingwedzi (South Africa) specimens. PCR amplification from the specimens produced an amplicon of ~ 550 bp, which is similar to the *An. rivulorum* size fragment. Lanes 1 and 11: 100 bp DNA ladder; lane 2: *An. funestus*; lane 3: *An. leesoni*; lane 4: *An. parensis*; lane 5: *An. rivulorum*; lane 6: *An. vaneedeni*; lane 7: negative control; lanes 8–10: three specimens from Shingwedzi

The ITS2 region of three suspected *An. rivulorum*-like specimens was amplified by PCR and produced an amplicon of ~ 550 bp (Fig. 3) which corresponds to the ITS2 region of *An. rivulorum*. Sequencing analysis of the ITS2 region from the specimens confirmed that the three specimens had a 100% identity. The established consensus sequence for the *An. rivulorum*-like from South Africa is:

>*An. rivulorum*-like (South Africa) GTTTAAACTCGGCCGATGCACACATTCTTGAGTGCCTACCAAATCCTTGATACACAAACACCTGACTACAGTGGTGCACGCGTGCAGCGAACTAAGCACTATGGCGAGACCCACGTCTAGTGTCGCTGAACCACACGCGCCTGCCCACTGTGCATAATGGCGTGCTCGGGAAGTAAAATTCTCGGGGGCGCTGAAGAGCGATGAGAGCATGGGGGCGGTACTCTGTTGCTGCCGGATCCCCCACTCCACGGGAGGCGGATGGTGCGTGTCTAGTTGCGTGTTGCGAACTCTGGCAGGACGTCCTGACAGCCCCGACAGCCCTGCATCAGGATTGTCGGCCTACTGTATCAGGGCCAAACGGCCGGCCAGGTCGCGTAATGCTCGCAGCTTAGACGTGCCACTCCGTCGCCATCGCACGAAAAACCGTAG.

There was a 99.5 and 99.3% sequence identity between the ITS2 region of the 3 specimens and the *An. rivulorum*-like from Eastern Zambia (GenBank accession number KR0148180) [24] and the *An. rivulorum*-like from the Southern province Zambia (GenBank accession number JN994147) [18]. The ITS2 region of the three specimens had a sequence identity of 97% with the ITS2 region from the *An. rivulorum*-like from Burkina Faso (GenBank accession number AF210725) [17]. The homology between the ITS2 region of the three specimens and that of *An. rivulorum* s.s. (GenBank accession number JN994148) [18] was 80%.

Females comprised 100% of the 64 *An. funestus* group captured, and ELISA results for *P. falciparum* were negative for all.

## Discussion

The primary vectors of malaria and their geographic distribution are well known, and control efforts directed at such primary vectors has achieved major successes in malaria reduction over the past decade [26]. The real challenge arises in the malaria elimination stages when traditional or standard methods of control need supplementation to address the remaining residual malaria transmission. In countries in the malaria elimination stage, such as South Africa, the causes of at least a portion of such residual malaria are not always clear, resulting in inexplicable small outbreaks or perpetuation of low levels of transmission which pose a threat to elimination targets and no small source of frustration. Secondary, or minor vectors, may be a contributing factor to such residual malaria transmission, especially when little is known regarding the presence or status of such secondary vectors, for example the recent finding in South Africa of *An. vaneedeni* naturally infected with *P. falciparum* [11]. It is for this reason that control programmes need to look wider during such critical phases of the final push to eliminate local transmission. *Anopheles rivulorum* is known to have the potential for low levels of transmission [13–15, 24], but nothing appears to be known regarding the vector status of *An. rivulorum*-like. This paper shows that *An. rivulorum*-like is fairly common in two areas of the Kruger National Park, north-eastern South Africa, where non-targeted, random collections of mosquitoes were made for non-malaria surveillance purposes.

The ITS2 region of the *An. rivulorum*-like from South Africa was more closely related to *An. rivulorum*-like from Zambia (99%) than the ITS2 region of *An. rivulorum*-like from Burkina Faso (97%). This divergence between the Zambian and Burkina Faso material was also noted by Norris and Norris [18] and they suggested it could be due to geographic variants. However, *An. longipalpis* type C and *An. vaneedeni* are seen as two distinct species, yet they show a 97.5% sequence similarity within the ITS2 region [27]. Without additional taxonomic, chromosomal and mating studies, it is impossible to predict if *An. rivulorum*-like from Burkina Faso and southern Africa are different species or mere geographical variants. With that being said, the sequence identity between the ITS2 region of the South African specimens and that of *An. rivulorum* was 80%, signifying that these specimens are more closely related to *An. rivulorum*-like than to *An. rivulorum*.

Greater effort should be directed towards establishing the potential role of this species in malaria transmission, with particular regard to residual malaria. The negative results for *P. falciparum* from female *An. rivulorum*-like reported in this paper are not surprising given that the collections were done inside the KNP, a strictly managed nature reserve. The KNP attracts almost exclusively middle to higher income tourists that are very unlikely to have infective gametocytes, therefore not representative of the situation in the malaria-endemic areas immediately adjoining this nature reserve, on both the South African and Mozambican sides. Interestingly, light trap collections by one of the authors (LB) in October 2017 along the Nwanedzi River (S22° 21.248′ E30° 35.434′) in Vhembe District, Limpopo Province, some 120 km northwest of Shingwedzi, yielded 52 *An. funestus* group members of which 48 were *An. rivulorum*, two *An. leesoni* and three failed PCR testing. This absence of *An. rivulorum*-like in a geographically close and environmentally similar setting, suggests either microhabitat differences in breeding preferences or seasonal variations in abundance.

In summary, *An. funestus* s.s. is known as one of the three most widespread and efficient vectors of malaria in Africa, with strong habits of endophagy, endophily and anthropophily. *Anopheles rivulorum* has been implicated in malaria transmission or found to harbour *P. falciparum* parasites in Kenya [15], Tanzania [13, 14] and Zambia [24]. *Anopheles vaneedeni* has been experimentally infected with *Plasmodium* in the laboratory [16] and recently found infected in nature [11]. As for the other members of the *An. funestus* group, there is one report [14] that possibly implicates *An. leesoni* and *An. parensis* in hosting *P. falciparum*, but not much appears to have been done to assess their role as secondary or minor vectors of malaria. The taxonomy/classification of *An. rivulorum*-like remains to be studied, that will require future comparative larval morphologies and whole genome sequencing. Formal naming of *An. rivulorum*-like is also required.

## Conclusion

The finding of *An. rivulorum*-like in South Africa now extends the range of this species to various points-of-presence straddling virtually the entire sub-Saharan region of Africa, with confirmed identifications in Burkina Faso, Cameroon, Zambia and South Africa.

With several African countries in the malaria elimination stage, and hopefully many more to reach this stage in the years ahead, the contribution of secondary malaria vectors becomes important. Little is known regarding the vector status of most members of the *An. funestus* group, other than *An. funestus* s.s. itself. At least one member, *An. rivulorum*, is known to host *P. falciparum*, and *An. vaneedeni* has been recently implicated. Two other species *An. parensis* and *An. leesoni* may also play a role as minor vectors. *Anopheles rivulorum*-like appears to be a fairly common species in parts of South Africa, a country which has ambitious targets for malaria elimination. The malaria vector capacity of *An. rivulorum*-like therefore needs to be assessed—as indeed do all other potential secondary vectors—especially in relation to the often unclear factors contributing to residual malaria transmission in a malaria elimination context.

## Abbreviations

CDC: Centers for Disease Control and Prevention
ELISA: enzyme linked immunosorbent assay
ITS2: internal transcribed spacer region 2
KNP: Kruger National Park
PCR: polymerase chain reaction
rDNE: ribosomal deoxyribonucleic acid
s.l.: sensu lato
s.s.: sensu stricto
